# Chinese expert consensus on the application of intravenous immunoglobulin in hematological diseases

**DOI:** 10.3389/fmed.2025.1544025

**Published:** 2025-04-01

**Authors:** Zhi Guo, Jie Zhu, Jun Wang, Liang Wang, Feifei Tang, Huiqiang Huang, Zhongjun Xia, Liqiong Liu, Danyu Wang, Nan Zhong, Huanhuan Zhou, Zhaogui Zhou, Wei Dai, Xiaojun Xu, Hao Zhou, Lijuan Deng, Jingye Meng, Zhiqiang Sun, Liang Shao, Yu J. Cao, Yansong Liu, Rong Qu, Guowei Li, Peng Chen, Hongyan Zhang, Jing Liang, Yuhua Li, Jiajun Liu, Zishan Xu, Soong Sung Inda, Xiaochen Xiang, Qingming Wu, Qiang Wang

**Affiliations:** ^1^Department of Hematology, The Sixth Affiliated Hospital of Shenzhen University Health Science Center, Shenzhen, China; ^2^Institute of Infection, Immunology and Tumor Microenvironment, Hubei Province Key Laboratory of Occupational Hazard Identification and Control, Medical College, Wuhan University of Science and Technology, Wuhan, China; ^3^Department of Hematology, Hongkong University Shenzhen Hospital, Shenzhen, China; ^4^Department of Hematology, Beijing Tongren Hospital, Capital Medical University, Beijing, China; ^5^Department of Hematology, Peking University People’s Hospital, Beijing, China; ^6^Department of Medical Oncology, State Key Laboratory of Oncology in South China, Guangdong Provincial Clinical Research Center for Cancer, Sun Yat-sen University Cancer Center, Guangzhou, China; ^7^State Key Laboratory of Oncology in South China, Department of Hematology, Sun Yat-sen University Cancer Center, Guangzhou, China; ^8^Department of Hematology, The Seventh Affiliated Hospital, Sun Yat-sen University, Shenzhen, China; ^9^Department of Hematology, Union Hospital, Tongji Medical College, Huazhong University of Science and Technology, Wuhan, China; ^10^Key Laboratory of Carcinogenesis and Translational Research (Ministry of Education/Beijing), Peking University Cancer Hospital & Institute, Beijing, China; ^11^Department of Hematology, National Clinical Research Center for Infectious Disease, Shenzhen Third People’s Hospital, Second Hospital Affiliated to Southern University of Science and Technology, Shenzhen, China; ^12^Department of Hematology, Shenzhen Hospital, Southern Medical University, Shenzhen, China; ^13^Department of Hematology, Zhongnan Hospital of Wuhan University, Wuhan, China; ^14^State Key Laboratory of Chemical Oncogenomics, Shenzhen Key Laboratory of Chemical Genomics, Peking University Shenzhen Graduate School, Shenzhen, China; ^15^Department of Critical Care Medicine, The Sixth Affiliated Hospital of Shenzhen University Health Science Center, Shenzhen, China; ^16^Department of Critical Care Medicine, Huizhou Central People Hospital, Huizhou, China; ^17^Department of Hematology, Huizhou Central People Hospital, Huizhou, China; ^18^Department of Hematology, The Fifth Medical Center of Chinese PLA General Hospital, Beijing, China; ^19^Department of Oncology, The Fifth Medical Center of Chinese PLA General Hospital, Beijing, China; ^20^Shandong Key Laboratory of Rheumatic Disease and Translational Medicine, Department of Oncology, Shandong Lung Cancer Institute, The First Affiliated Hospital of Shandong First Medical University & Shandong Provincial Qianfoshan Hospital, Jinan, China; ^21^Hematology Department, Southern Medical University, Zhujiang Hospital, Guangzhou, China; ^22^Guangdong Engineering Research Center of Precision Immune Cell Therapy Technology, Guangzhou, China; ^23^Department of Hematology, The Third Affiliated Hospital, Sun Yat-sen University, Guangzhou, China; ^24^Department of Hematology, Pamela Youde Nethersole Eastern Hospital, Chai Wan, Hong Kong SAR, China; ^25^Department of Clinical Oncology, Pamela Youde Nethersole Eastern Hospital, Chai Wan, Hong Kong SAR, China

**Keywords:** IVIG, clinical application, hematologic disorders, chimeric antigen receptor T-Cell, hematopoietic stem cell transplant (HSCT), expert consensus

## Abstract

Intravenous immunoglobulin (IVIG), first developed for the treatment of patients with antibody deficiencies, is now widely used in clinical practice, especially in hematological and immune system diseases, and its application in hematological oncology chemotherapy, cellular immunotherapy and hematopoietic stem cell transplantation (HSCT) is becoming more and more common. The Chinese Collaborative Group for Infection Immunology and Microecology Research Translation Collaborative Group organized relevant experts to discuss and propose the “Chinese expert consensus on the application of intravenous immunoglobulin in hematological diseases,” which was formulated based on the progress of research on the application of IVIG in blood diseases, and provides a basis for the standardization of the use of IVIG in hematologic disorders.

## 1 Introduction

Intravenous immunoglobulin (IVIG) is a blood product obtained from the plasma of healthy donors, consisting primarily of polyclonal immunoglobulin G (IgG). It has anti-inflammatory and immunomodulatory effects, and was first introduced in the early 1980s for the treatment of primary humoral immunodeficiency (1). At present, IVIG has been widely used in clinic, such as the treatment of immune system diseases (including systemic lupus erythematosus, Kawasaki disease, primary immunodeficiency, etc.), hematologic disorders (including immune thrombocytopenia, autoimmune hemolytic anemias, hemolytic disease of the newborn, etc.) and neurological disease (including Guillain-Barre syndrome, chronic inflammatory demyelinating polyneuropathy, myasthenia gravis, polymyositis, multiple sclerosis and autoimmune encephalitis) (2–5). Its application in chemotherapy, targeted therapy, cellular immunotherapy and HSCT for malignant hematological tumors is also becoming more prevalent (6–8). In order to further improve the standardized application of IVIG in hematological diseases, the Chinese Collaborative Group for Infection Immunology and Microecology Research Translation Collaborative Group has organized a multi-disciplinary team (MDT) steering committee composed of experts from hematology (including HSCT), infectious diseases, critical care medicine, pharmacy and other specialties to discuss the issue, and to synthesize the current status of related research at home and abroad to formulate expert recommendations for the standardized application of IVIG in hematological diseases. The expert recommendation on the application of IVIG in hematological diseases was formulated based on the progress of domestic and international research on the application of IVIG in hematological diseases, which provides a basis for the standardization of the use of IVIG in hematological diseases. This expert recommendation uses the GRADE/DECISION Evidence to Decision Making Framework to determine the direction and strength of the recommendation, with the GRADE methodology assessing the quality of evidence rated as high (A), moderate (B), low (C), or very low (D). Based on the GRADE evidence the MDT panel rated the strength of the recommendation as strong and weak recommendation (for or against interest intervention), or not recommended if the overall quality of the evidence in the key endpoints was very low, and ultimately the full MDT experts voted and reached consensus. Strength of evidence recommendations and level of evidence criteria in treatment guidelines (Table 1).

**Table 1:** Strength of evidence recommendations and level of evidence criteria in treatment guidelines.

Level of evidence standards	
Grade A	Meta-analysis or systematic review of multiple randomized controlled trials; Multiple randomized controlled trials (RCT) or one RCT with sufficient sample size
Grade B	At least one high-quality RCT
Grade C	Controlled trials that are not randomized but are well-designed; or well-designed cohort or case–control studies
Grade D	Case series without concurrent controls or expert consensus
Strength of evidence recommendations	
Class I	If a randomized controlled trial cannot be done, strong recommendation was defined when > 85% of panelists, agreed with a statement
Class II	If a randomized controlled trial cannot be done, weak recommendation was defined when 75–85% of panelists, agreed with a statement
Class III	If a randomized controlled trial cannot be done, no recommendation was defined when < 75% of panelists, agreed with a statement

## 2 Overview of IVIG

### 2.1 Mechanism of IVIG

**Figure 1:**
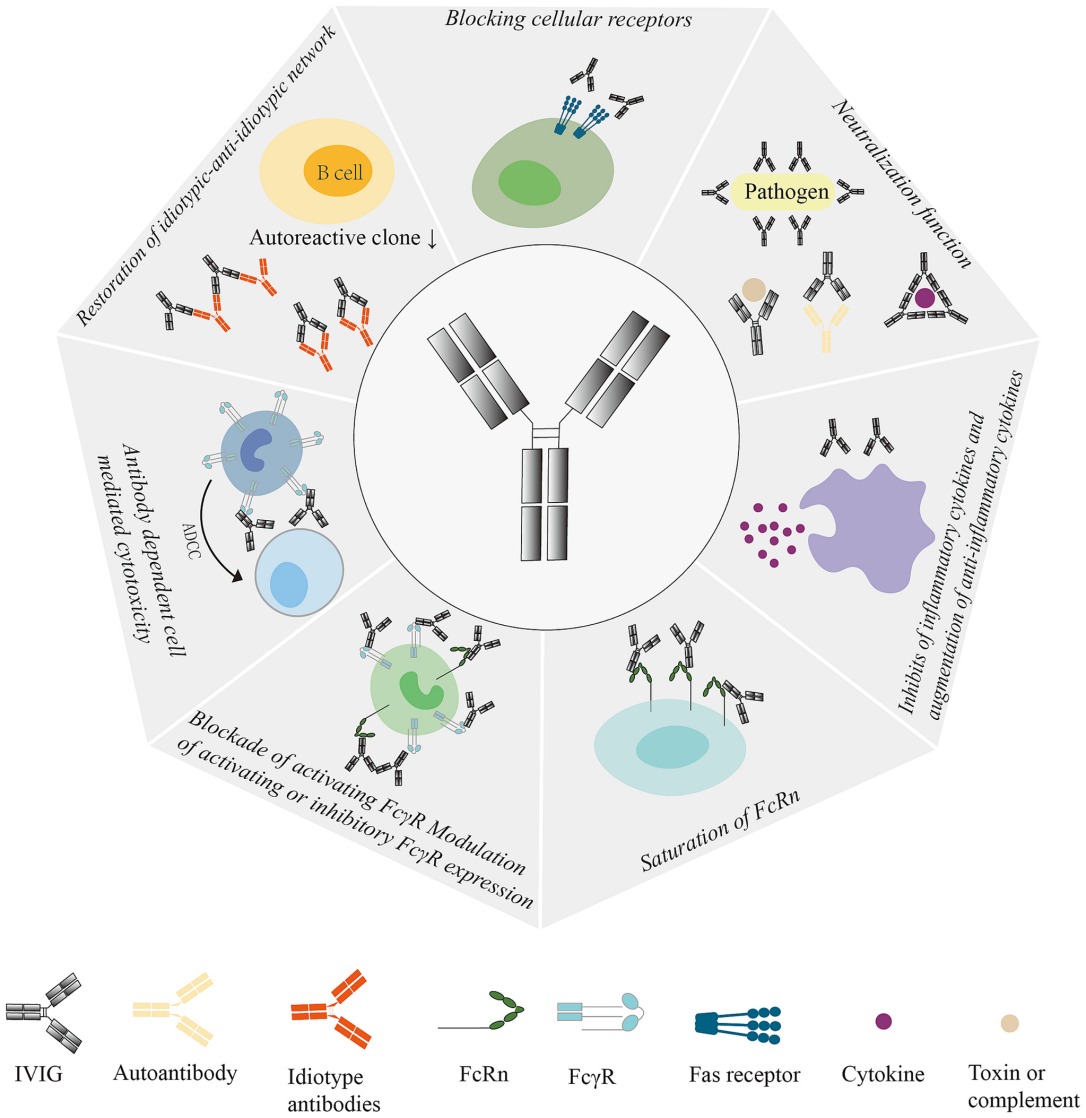
Mechanism of action of IVIG: Regulates Fc receptor expression and function, interferes with complement activation and regulates cytokine secretion, delivers anit-idiotype antibodies, neutralizes bacterial toxins, and regulates dendritic cell, T-cell, and B-cell activation, differentiation, and their effector functions. ADCC, antibody-dependent cell-mediated cytotoxicity; IVIG, Intravenous Immunoglobulin; Fas, Factor-related Apoptosis; FcRn, neonatal Fc receptor; FcγR, IgG Fc receptor.

IVIG is used as a therapeutic substitution in primary and secondary immunodeficiencies as well as an immunomodulatory agent. IVIG has many immunomodulatory effects, however the exact mechanism for of this is not completely clear. At present, the mechanisms of IVIG include: (1) the Fc fragment of IgG specifically binds to some immune effector cells, closes the monocyte macrophage Fc receptor, and inhibits antibody-dependent cell-mediated cytotoxicity. (2) IVIG plays an immunosubstitutive role by binding to antigens in the F(ab)_2_ fraction. A variety of specific antibodies in IVIG can either directly seal off the site of action of the organism’s antigens, resulting in a decrease in the titer of pathogenic antibodies, or bind to the organism’s antigens to form an antigen–antibody complex that is phagocytosed by the phagocytes. (3) IVIG has a broad spectrum of anti-normal human protein and anti-idiotypic antibody, which can accelerate the clearance and neutralization of circulating autoantibodies by seizing the action site of autoantibodies. (4) Anti-inflammatory effect: IVIG can regulate the secretion of various cytokines and inhibit the production of pro-inflammatory cytokines, thus achieving anti-inflammatory effect. In addition, IVIG may also exert potential anti-inflammatory effects through complement inhibition, blockade of Fas ligand-mediated apoptosis, and other mechanisms, and these mechanisms are not mutually exclusive but synergistic. (5) Eliminating pathogenic microorganisms: IVIG contains a variety of anti-bacterial toxin antibodies, neutralizing superantigens contained in bacterial toxins, and can eliminate pathogenic microorganisms such as viruses and bacterial toxins that persist in the body (9–12). (6) FcγRIII regulates dendritic cell properties: IVIG enhances the production of IL-1 and inhibits the production of IL-12 by inhibiting the differentiation and maturation of DCs (13) (Figure 1).

### 2.2 Adverse reactions

The risk of infectious complications from IVIG is extremely low. The requirements for donor screening and infectious disease testing for imported plasma are very stringent. In addition, at least one step in the IVIG manufacturing process must remove the enveloped virus, while at least two complementary viral inactivation methods must be used to prevent infectious pathogens that may be present during the screening process. Recently, significant advances have been made in the way IVIG is produced, reducing the likelihood of the presence of infectious pathogens while ensuring safety and that side effects are minimized (14). However, IVIG, a blood product isolated from the combined plasma of thousands of healthy blood donors, still carries the risk of infectious agent transmission (15). There are few reports on the prospective data of IVIG-related adverse reactions. The overall incidence of IVIG infusion-related reactions is between 3 and 15%, and these reactions are usually phlogistic in nature and self-limited (16). IVIG related reactions are headache, nausea, vomiting, fever, rash, etc. Mild to moderate adverse reactions can usually be alleviated or avoided by slowing down the infusion speed of IVIG or stopping the infusion. Patients with multiple reactions are given antipyretics and antihistamines in advance. Contraindications to IVIG include individuals who are allergic to human immunoglobulin and individuals with selective IgA deficiency with anti-IgA antibodies. Serious adverse reactions are severe allergic reactions, acute renal failure, thromboembolic events, aseptic meningitis occurrences, neutropenia, autoimmune hemolytic anemia and rare events of arthritis. In view of the lack of data on the severity and incidence of their potential adverse events, this Expert Advice concludes that clinicians should limit the prescription of IVIG and use it only when there is sufficient evidence (17), and since cost is a limiting factor as well as the huge cost of IVIG.

## 3 Application of IVIG in hematologic disorders

### 3.1 Immune thrombocytopenia

The earliest discovery of the efficacy of high-dose IVIG was in the treatment of primary immune thrombocytopenia (ITP) (18). ITP is an autoimmune disorder characterized by a decrease in platelet count due to destruction of platelets by the immune system. ITP treatment guidelines mention that the first line treatment of choice is glucocorticoids and IVIG (19). ITP was the first autoimmune disease to be treated with IVIG, which is as effective as corticosteroid treatment (20, 21). In cases of active bleeding or when corticosteroids are contraindicated in patients, IVIG can induce a rapid increase in platelet count and quickly stop bleeding. The effect begins 1–3 days and the peak lasts for 2–7 days (22). Different centers use different IVIG doses and regimens. A meta-analysis showed that there were no significant differences in clinical outcomes and progression to chronic ITP with low-dose IVIG (<2 g/kg) versus high-dose IVIG (>2 g/kg), and that low-dose IVIG had fewer adverse effects and was less costly (23). The American Society of Hematology guideline panel recommends a single dose of IVIG 0.8 to 1 g/kg or a short course of glucocorticoids as first-line treatment for ITP, but both can be used in combination in order to rapidly elevate platelet levels (24). Patients with ITP treated with IVIG must have weekly complete blood counts (CBC) to assess efficacy and duration of therapy. In addition, patients should be informed that headache due to aseptic meningitis may occur after administration (25).

**Expert recommendation 1**: For adult patients with acute ITP requiring treatment, the recommended first-line treatment is a single dose of IVIG 0.8–1.0 g/kg or 0.4 g/kg/d for 3 to 5 days, with repeated administration if necessary. For emergency treatment of patients with hemorrhage, glucocorticoids in combination with IVIG 1 g/kg/d for 2 days are recommended (Class I).

### 3.2 Hemophagocytic syndrome

Hemophagocytic syndrome (HPS), also known as hemophagocytic lymphohistiocytosis (HLH), is a syndrome of excessive inflammatory response caused by abnormal activation and proliferation of the lymphocyte, monocyte and macrophage systems, and secretion of large amounts of inflammatory cytokines, causing a series of inflammatory reactions. Depending on the etiology, HLH is categorized as primary and secondary. Primary HLH results in defective cytotoxic function of natural killer cells and T lymphocytes. Secondary HLH can be triggered by infection, malignancy, autoimmunity, etc. It is considered to be caused by temporary acquired immunodeficiency leading to defective NK cells, and the clinical manifestations are characterized by persistent fever, hepatosplenomegaly, pancytopenia, and hemophagocytosis found in bone marrow, liver, spleen, and lymph node tissues. The HLH-1994 regimen is suitable for all types of HLH First-line induction therapy, including etoposide and dexamethasone (26–28). The application of IVIG can inhibit the activity of macrophages and reduce the damage of tissues and cells through various anti-inflammatory mechanisms. At present, IVIG can be considered as an auxiliary treatment for HLH, especially in the early stage of infection-related HLH. Some patients show a good response to IVIG alone, and the treatment with IVIG can avoid the adverse reactions of other treatment medications. IVIG should be supplemented with treatment of infection and criteria for hematopoietic stem cell transplantation in accordance with treatment guidelines (29–31).

**Expert recommendation 2**: IVIG 0.4 g/kg/week can be added as co-infection supportive therapy or neutropenia co-infection in patients with HLH (Class I).

### 3.3 Thrombotic thrombocytopenic purpura

Thrombotic Thrombocytopenic Purpura (TTP) is a severe diffuse thrombotic microangiopathy characterized by microangiopathic hemolytic anemia, consumptive reduction of platelets, and organ damage (e.g., kidneys, central nervous system, etc.) due to microthrombosis. The pathogenesis of TTP mainly involves factors such as lack of activity of von Willebrand factor (vWF) lyase (ADAMTS13), abnormal release of vascular endothelial cell vWF, abnormal activation of complement, and abnormal activation of platelets, leading to microvascular thrombosis, microvascular hemolysis, and subsequent organ ischemia, hypoxia, and dysfunction, resulting in clinical symptoms of TTP pentad syndrome, namely thrombocytopenic purpura, microvascular hemolysis, central nervous system symptoms, fever, and kidney damage. Most TTP patients have a sudden onset and dangerous condition, with a mortality rate of up to 90% if left untreated. High-dose immunoglobulin may be effective in some patients who fail plasma exchange by inhibiting platelet aggregation and splenic destruction of platelets and red blood cells (32).

**Expert recommendation 3**: High-dose IVIG is recommended for patients with recurrent or refractory TTP, at a dose of 1.0 g/kg/d for 2 days or 0.4 g/kg/d for 5 days. If necessary, it can be given repeatedly, but the therapeutic effect may not be as good as plasma exchange (Class II).

### 3.4 Acquired hemophilia A

Acquired Hemophilia A (AHA) is an acquired hemorrhagic disease characterized by a decrease in FVIII activity (FVIII: C) due to the production of specific autoantibodies that inhibit FVIII in the body. AHA is often associated with severe life-threatening bleeding, and subcutaneous hematomas are a characteristic manifestation of AHA, in addition to muscle, joint, gastrointestinal, and vaginal bleeding. Effective hemostasis is achieved with correct diagnosis and prompt treatment. The principles of treatment include initial hemostatic therapy (bypass, and activated human or activated porcine FVII are the current standard of care) and etiologic therapy (human or porcine FVIII), which depends on the severity of the hemorrhage and the characteristics of the antibodies (33, 34).

**Expert recommendation 4**: In patients with AHA who do not respond to immunosuppressive regimens, the administration of IVIG 1.0 g/kg/d for 2 days or 0.4 g/kg/d for 5 days is recommendedIVIG has poor efficacy in the AHA, and therefore its use for the purpose of blocking autoantibodies to FVIII is not recommended (Class II).

### 3.5 Autoimmune hemolytic anemia

Autoimmune hemolytic anemia (AIHA) is a type of hemolytic anemia caused by disorders in the regulation of the body’s immune function, leading to the production of autoantibodies attached to the surface of red blood cells accelerating the destruction of red blood cells through the antigen–antibody reaction, typically mediated by complement activation. The production of autoantibodies involves many links of the immune system, such as the cross reaction between endogenous red blood cells and exogenous/environmental antigens, acquired factors such as infection and malignant tumors, structural change of auto-antigens and the disorder of antigen presentation, and the dysfunction of B cells and T cells. AIHA is usually categorized based on a positive direct antiglobulin test (DAT) and the optimal temperature required for the antibody to act on the erythrocyte membrane (hot, cold, and mixed forms), with the IgG-mediated warm-antibody phenotype being the most common. First line treatment for AIHA consists of glucocorticosteroids or glucocorticosteroids in combination with rituximab. For patients who fail glucocorticoid therapy, relapse, intolerant and dependent, second-line therapy is administered and the preferred regimen is rituximab. Third-line treatment includes splenectomy and immunosuppressive agents such as cyclosporine A, sirolimus, and azathioprine. AIHA may occur secondary to diseases that may trigger autoantibody production, such as chronic lymphocytic leukemia and systemic autoimmune diseases. In addition, the incidence of AIHA after HSCT is increasing (35). In common variable immunodeficiency, disease-associated warm antibody-type secondary AIHA, maybe present in some patients with this condition. In addition to the risk of infection caused by the underlying immunodeficiency itself, such patients are further prone to severe and life threatening infections especially after glucocotricoids, immunosuppressants or rituximab, and the administration of intravenous immunoglobulin is recommended to elevate the level of immunoglobulin during the course of treatment and reduce the risk of infection (36).

**Expert recommendation 5**: High-dose IVIG can be used for life-threatening hemolysis or hemolysis for which other treatments are ineffective, and it is recommended that high-dose corticosteroids be given in combination with IVIG 1.0 g/kg/d for 2 days or 0.4 g/kg/d for 5 days as salvage therapy only in cases of severe or rapid hemolysis (Class I).

### 3.6 Hypogammaglobulinemia

Hypogammaglobulinemia (HG) is an immune system disorder, defined as serum IgG < 7 g/L, which results in lower antibody serum levels and an increased risk of infection due to the failure of the immune system to produce enough immunoglobulin (37). HG may be caused by various underlying primary/congenital immune system defects or secondary immune deficiency states (such as hematological malignancies, protein loss diseases, etc.). Primary humoral immune deficiencies are most commonly X-linked agammaglobulinemia (XLA) and common variable immune deficiencies. The most common clinical features of HG are recurrent bacterial infections, malabsorption syndrome, steatorrhea, protein-losing enteropathy, etc. Primary immunodeficiencies may be associated with a variety of autoimmune diseases, such as AIHA, ITP, dermatomyositis, etc., and malignant tumors (38, 39).

**Expert recommendation 6**: When complications related to primary immunodeficiency, such as infection, occur in HG patients, prophylactic treatment IVIG infusion, IVIG 0.4 g/kg/dose, is used, recommended every 3 weeks typically for the remainder of the patient’s life.

## 4 Application of IVIG in the treatment of hematological tumors

### 4.1 Chimeric antigen receptor T-cell immunotherapy

Chimeric antigen receptor T-Cells (CAR-T) are genetically engineered to integrate gene fragments of single-chain variable regions and co-stimulatory molecules targeting tumor antigens into the T-cell genome and express them on T-cells, which specifically recognizes tumor antigens and initiates the downstream signaling pathway to proliferate, activate, and exert a CAR-T cell targeted tumor-killing effect, and the common targets of CAR-T cell therapy for refractory/relapsed acute B-lymphoblastic leukemia are CD19 and CD22 (40). CAR-T cell products have been used to achieve good efficacy in the treatment of relapsed refractory B-cell tumors, with CD19 as the main target, and the indications mainly include diffuse large B-cell lymphoma, condylomatous cell lymphoma, and follicular lymphoma (41). CAR-T cell in the treatment of relapsed and refractory multiple myeloma is targeted at B cell maturation antigen (BCMA), and many other CAR-T targets such as CS1 (CS1 is a cell surface glycoprotein of the signaling lymphocyte activation molecule (SLAM) receptor family), G protein-coupled receptor class C group 5 member D (GPRC5D), CD38 and CD138 have also entered clinical trials (42). Pretreatment chemotherapy regimens prior to infusing CAR-T cells back into the body can cause lymphocyte exhaustion (43), CAR-T cell therapy also destroys normal B cells, and the majority of patients receiving CAR-T therapy have varying degrees of hypogammaglobulinemia or B-cell deficiencies (44, 45). This along with other factors may lead to increased risk of infections after CAR-T therapy. After receiving BCMA CAR-T cell therapy, 76% of patients with multiple myeloma developed hypogammaglobulinemia, increasing the risk of infection (46). The incidence of infection within 28 days after reinfusion of CAR-T in patients with acute lymphoblastic leukemia was 40% (47), while in another clinical trial of BCMA CAR-T cell therapy for multiple myeloma, the incidence of infections ranged from 42 to 69% (48). The consensus among Chinese experts is that prophylactic intravenous immunoglobulin (IVIG) is a routine adjuvant therapy for patients receiving CAR-T cell therapy. National Comprehensive Cancer Network (NCCN) guidelines recommend that patients receiving CAR-T cell therapy should be regularly supplemented with immunoglobulin infusions. The European Society for Hematology and Bone Marrow Transplantation and the American Society for Hematology and Bone Marrow Transplantation recommend that IVIG replacement therapy after CAR-T therapy should follow the X-linked absence of gammaglobulinemia principle (48).

**Expert recommendation 7**: After CAR-T cell therapy, the number of B lymphocytes and immunoglobulin level should be checked regularly, and IVIG infused at least once a month, at a dose of 0.4 g/kg/dose, until immunoglobulins and B cells returned to normal range or 6 months after CAR-T cell therapy (Class I).

**Expert recommendation 8**: Patients with serum IgG < 4 g/L and severe or recurrent infections after CAR-T cell therapy should continue to receive IVIG infusion at least once a month at a dose of 0.4 g/kg/dose until the risk factors are eliminated. If the serum IgG level is 4–6 g/L and there is still severe or recurrent infections after treatment, IVIG infusion should be given at least once a month at a dose of 0.4 g/kg/dose. If the serum IgG is greater than 6 g/L and there are recurrent infections, it is recommended to further evaluate the levels of the types of immunoglobulins (IgG, IgA, IgM and IgE) and the number of B cells (Class I).

### 4.2 Chemotherapy for acute leukemia

Intense chemotherapy is the main method for patients with acute leukemia (AL) to alleviate their illness and prolong their survival. However, AL supplemented is affected by various factors such as severe granulocyte deficiency after chemotherapy and increased risk of infection associated with the use of immunosuppressants and glucocoticoids causing significant economic burden and increased morbidity and mortality. Therefore, it is extremely important to prevent and actively control the occurrence of infection after AL chemotherapy. Although B cells are affected and immunoglobulin levels maybe decreased in AL patients receiving chemotherapy, IVIG infusion has not been widely and systematically studied for the prevention or treatment of related infections in AL patients. Most infections are due to the disease itself and/or chemotherapy-related neutropenia, and for patients who are in remission and have completed treatment, the immune deficiency may last for 6–12 months after the treatment is completed (49). When neutropenia occurs after AL chemotherapy, the inflammatory response mediated by neutrophils is not significant. Severe neutropenia most commonly occurs during hematopoietic stem cell transplantation, AL initial induction chemotherapy, and high-dose chemotherapy consolidation stage. It is necessary to identify neutropenic fever early and start empirical systemic antimicrobial therapy in a timely manner to avoid sepsis and death. It is important to administer IVIG simultaneously with anti-infective therapy (50).

**Expert recommendation 9**: Individualized assessment of the benefits and risks of IVIG infusion in patients with AL is recommended, and after weighing the risks and costs associated with treatment, at least one IVIG infusion per month is recommended at IVIG 0.4 g/kg/dose, with a target serum IgG of 4 to 6 g/L, and in the presence of breakthrough infections, a serum IgG > 6 to 8 g/L. Serum IgG levels, specific antibody titers and infection pattern are used to guide the IVIG treatment (Class II).

AL-intense chemotherapy can also cause severe myelosuppression, and repeated transfusion therapy is required after chemotherapy. Repeated transfusions can lead to a number of adverse transfusion reactions and platelet ineffective transfusion can occur in some patients with repeated platelet transfusion, which is mainly attributed to the development of specific antibodies against human leukocyte antigens. AL can also cause a decrease in ABO blood type antigens in patients, affecting clinical blood matching, increasing the risk of hemolytic reactions due to blood type incompatibility, and even leading to patient death in severe cases. The application of IVIG can block the Fc receptors of monocytes and macrophages, reducing the destruction of tissue cells mediated by allogenic antibodies and thereby, reducing adverse reactions to blood transfusion, and improving safety.

**Expert recommendation 10**: For AL patients with ineffective repeated platelet transfusions and obvious adverse reactions to blood transfusion, IVIG before transfusion can be recommended at a dose of 0.2–0.4 g/kg/dose (Class II).

### 4.3 Treatment of multiple myeloma

Infection remains a major cause of morbidity and mortality in patients with multiple myeloma (MM) due to the cumulative effect of disease, treatment and host-related factors. Disease-related plasma cell abnormalities, the effects of antitumor therapy, older age of onset and disease-related complications (e.g., renal failure) all contribute to an increased susceptibility to infections in patients with myeloma, and therefore the prevention of infections during the treatment of MM is of utmost importance. Prevention of infection during the process is crucial, and optimal prevention strategies include antimicrobial prophylaxis, infection control measures, and IVIG infusion in some patients (51). The NCCN guidelines mention that the risk of infection in MM patients is associated with treatments such as autologous HSCT, bispecific antibodies, CAR-T cell therapy, cytotoxic chemotherapy drugs, proteasome inhibitors, anti-CD38 monoclonal antibodies, glucocorticoids, etc. 75% of patients receiving bispecific antibody therapy in the MajesTEC-1 study developed hypogammaglobulinemia (52). An earlier study retrospectively analyzed 3,000 MM patients showed 10% died within 60 days, 45% of whom died of infections (53). It has also been shown that myeloma patients receiving IVIG prophylaxis had fewer cases of serious infections than patients who did not receive IVIG, and that prophylactic use of IVIG reduced the risk of serious infections by 90% in MM patients treated with bispecific antibodies to BCMA (54, 55). Therefore, 0.4 to 0.6 g/kg IVIG infusion every month for 6–12 months is beneficial to MM patients, and the dosage and time should be adjusted according to the patient’s condition to ensure full prevention of infection (56). IVIG has a major limitation: it accelerates immunoglobulin metabolism in people with high levels of myeloma protein. There are two points to note when administering IVIG: (1) Immune modulators such as thalidomide, lenalidomide, and pomalidomide increase the risk of thrombosis during treatment, and IVIG infusion can further increase the risk of thrombosis (57). (2) Some MM patients may experience renal dysfunction, and IVIG infusion may further increase the risk of renal injury. Currently, most IVIG products remove sucrose (previously used as a stabilizer), which can reduce the risk of IVIG renal injury (58).

**Expert recommendation 11**: For MM patients with serum IgG ≤4 g/L and severe or recurrent infections, at least one IVIG infusion of 0.4 g/kg/dose per month is recommended, and for MM patients after CAR-T cell therapy, at least one IVIG infusion of 0.4 g/kg/dose per month is recommended until 1 year after the end of CAR-T therapy (Class II).

### 4.4 Treatment of non-Hodgkin lymphoma

Non-Hodgkin’s lymphoma (NHL) is the most common malignant tumor of lymphatic system, among which B cell-derived lymphoma accounts for more than 85% of the cases (59). Chemotherapy can effectively treat lymphoma, but chemotherapy not only leads to bone marrow suppression, but also has an immunosuppressive effect. CD20 monoclonal antibody is a major breakthrough in the treatment of B-cell lymphoma. However, CD20 monoclonal antibody in combination with chemotherapy leads to a further decrease in the immune function of the patient, predisposing to a variety of infections, such as herpes zoster, lung infections and others. CD20 monoclonal antibody kills abnormal and normal B lymphocytes at the same time, which reduces the number of B lymphocytes in peripheral blood and antibody production, eventually leading to humoral immune deficiency and increasing the risk of infection in NHL patients (60). The use of targeted drugs (such as Bruton’s tyrosine kinase inhibitors), immune modulators (such as lenalidomide), immune checkpoint inhibitors, and other drugs can also increase the incidence of infection (61, 62). The guidelines of the British Hematology Standards Committee suggest that NHL patients should receive regular IVIG infusions during treatment to reduce the occurrence of infections (63), while the NCCN guidelines recommend that NHL patients receiving CD20 monoclonal antibody and CAR-T cell therapy should receive regular IVIG infusions.

**Expert recommendation 12**: NHL patients need regular infusion of IVIG to enhance immunity and prevent infection during treatment, especially for NHL patients with IgG ≤ 4 g/L, recurrent or severe infection, using IVIG infusion every 3–4 weeks, IVIG 0.4 g/kg/dose (Class II).

## 5 Application of hematopoietic stem cell transplantation

HSCT, especially allogenetic stem cell transplantation (allo-HSCT), is still the only cure for many benign and malignant hematological diseases (64, 65). Meanwhile, post-transplant complications such as infection, graft-versus-host disease (GVHD), thrombotic microangiopathy, and sinusoidal obstruction syndrome (SOS), also know as Veno-occlusive Disease (VOD) are closely related to transplantation-related mortality, and these complications have hindered transplantation development (66, 67). Human immunoglobulin has synergistic anti-infection and immune regulation functions, and is often used to control infection and complications such as GVHD after transplantation. After HSCT, IVIG can effectively prevent cytomegalovirus (CMV) infection, GVHD, bacterial infection, etc. The FDA of the United States approved IVIG as a preventive drug for patients with HSCT over 20 years old to reduce the incidence of infection complications such as pneumonia (11).

### 5.1 Application in post-transplant CMV infection

CMV infection is one of the main causes of infection and poor prognosis in patients undergoing allogeneic HSCT. CMV can lead to CMV disease, acute and chronic GVHD, opportunistic infection, bone marrow suppression and other serious adverse events, affecting the prognosis of patients undergoing HSCT. The incidence of CMV disease ranges from 10 to 40%, with CMV pneumonia being the most predominant type, with a mortality rate as high as 70%. Due to the wide application of prevention and preemptive treatment, the incidence of CMV disease has been reduced to less than 10%, with a case fatality rate of about 20 to 60% (68). The reactivation of CMV within 100 days after transplantation is accompanied by the increase of transplant-related mortality. The preemptive Treatment with antiviral drugs and the use of CMV-negative or leukocyte-depleted blood products have greatly reduced the incidence of CMV infection after transplantation (69). Early studies showed that preventive application of IVIG reduced CMV infection rate and mortality of interstitial pneumonia without CMV infection (70). IVIG combined with ganciclovir significantly changed the prognosis of patients with CMV pneumonia after transplantation. However, Schmidt et al. gave prophylactic IVIG to patients at high risk of CMV disease after allograft transplantation and showed no significant difference in the rate of CMV infection, or in the cumulative incidence of GVHD, when compared to a control group that was not given prophylactic IVIG (70). Another study evaluated the weekly preventive use of IVIG 5 g from −7 days to +98 days after transplantation. The results showed that the cumulative incidence of CMV infection was similar between the subjects treated with IVIG and the control group at +100 days after transplantation. These data showed that IVIG had no obvious advantage for CMV reactivation in transplant patients who could receive antiviral drugs. However, whether the high-risk population of CMV reactivation can benefit from these interventions has not been confirmed by research (71).

**Expert recommendation 13**: It is recommended that IVIG can be used prophylactically in patients with CMV high-risk activators in allo-HSCT, with 1 to 2 weekly infusions of IVIG, IVIG 5 g to 10 g/dose, up to 100 days post-transplantation (Class II).

### 5.2 Application in GVHD after transplantation

At least 50% or more of transplant related deaths are directly or indirectly related to GVHD, and effective prevention and treatment methods for GVHD need to be sought. GVHD is often closely related to delayed immune function reconstruction in transplant patients, and promoting the formation of immune reconstruction is the fundamental way to solve complications after allo HSCT. Induced immune tolerance means that after transplantation the recipient is resistant to rejection and the graft maintains stable function for a long time (72). The research hotspots in recent years mentioned that the role of intestinal flora in the body’s anti-tumor immune response including chemotherapy and allo-HSCT has received increasing attention. The gut microbiota plays an important role in the pathological and physiological processes of GVHD (73). Changes in gut microbiota may determine the severity of GVHD. After transplantation, the composition of gut microbiota is altered, leading to dysbiosis. Gut microbiota can easily penetrate damaged intestinal mucosa, causing abnormal immune responses, activating T lymphocytes, promoting the release of inflammatory mediators, and causing damage to the gastrointestinal mucosal barrier, thereby damaging target organs such as the gastrointestinal tract (74–76). Based on the immunomodulatory effect of IVIG, a large randomized controlled study was conducted on allo-HSCT patients, comparing the weekly administration of IVIG 0.5 g/kg from −7 days to +90 days after transplantation, followed by monthly administration of IVIG 0.5 g/kg from 90 days to 360 days. Multivariate analysis showed that compared with IVIG recipients, the control group had an increased risk of acute GVHD > grade 2 (RR 1.63, *p* < 0.0056) (77). In another study, more than 600 subjects received 0.1 g/kg, 0.25 g/kg or 0.5 g/kg IVIG at random, once a week until the 90th day after transplantation, and then once a month until 1 year after transplantation. The study showed that the incidence of acute GVHD was the lowest in the HLA-matched unrelated donor group and the subjects who received the highest dose of IVIG (78).

**Expert recommendation 14**: IVIG 0.5 g/kg per week from pre-transplantation −7 days to post-transplantation day 90 and 0.5 g/kg per month from post-transplantation day 90 to 1 year post-transplantation is recommended for those at high risk for GVHD when mismatched with allo-HSCT (Class II).

### 5.3 Application in post-transplant bacterial infections

HSCT patients undergo myeloablative chemotherapy. After ultra-intensive pretreatment and radiotherapy, the patients are in the ablative period of bone marrow, and the process of immune function reconstruction is longer, with longer period of severe neutrophil deficiency. In addition, the incidence of various infections such as bacteria, fungi and viruses is higher after long-term use of immunosuppressants such as corticosteroids. Regarding whether IVIG is needed to prevent bacterial infection during transplantation, the relevant guidelines of the American Society of Blood and Bone Marrow Transplantation unanimously recommend that IVIG should not be routinely used to prevent bacterial infection after transplantation (79). However, when the patient is complicated with severe hypogammaglobulinemia (serum IgG < 4 g/L), it is suggested to receive preventive IVIG infusion from the beginning of pretreatment chemotherapy before transplantation to 100 days after transplantation to maintain serum IgG > 4 g/L. There is a lack of sufficient randomized controlled studies to support this recommendation, and the only objective data are based on IVIG pharmacokinetic studies showing a half-life of approximately 6 days in transplanted patients and 22 days in normal subjects, and the explanation for this discrepancy may be the increased proteolytic metabolism and reduced protein conversion and synthesis due to GVHD (80).

**Expert recommendation 15**: IVIG 0.5 g/kg per week is recommended for patients with serum IgG <4 g/L from 7 days pretransplant to 90 days posttransplant, and 0.5 g/kg per month from 90 days posttransplant to 1 year posttransplant, so as to prevent serious bacterial infection. Serum IgG concentration should be monitored every 2 weeks, and individualized treatment should be carried out according to serum IgG level and infection (Class II).

## 6 Conclusion

This consensus is based on the existing evidence both domestically and internationally, as well as the careful discussions organized by the Chinese Infection Immunology and Microecology Research Translation Collaborative Group with relevant experts. Based on the evidence grading method, 15 suggestions were proposed for the application of IVIG in the treatment of hematological diseases, hematological tumors, and hematopoietic stem cell transplantation (Table 2). It is hoped to serve as a reference for clinicians and help standardize the dosage of IVIG used to treat hematological diseases.

**Table 2:** 15 recommendations were proposed for the application of IVIG in the treatment of hematological diseases, hematological tumors, and hematopoietic stem cell transplantation.

Disease	Uses and dosage	Strength of evidence recommendation and expert agreement
Application of IVIG in hematologic disorders		
ITP	For adult patients with acute ITP requiring treatment, the recommended first-line treatment is a single dose of IVIG 0.8–1.0 g/kg or 0.4 g/kg/d for 3 to 5 days, with repeated administration if necessary.	Class I; 100%
	For emergency treatment of patients with hemorrhage, glucocorticoids in combination with IVIG 1 g/kg/d for 2 days is recommended.	
HPS	IVIG 0.4 g/kg/week can be added as co-infection supportive therapy or neutropenia co-infection in patients with HLH.	Class I; 87.9%
TTP	High-dose IVIG is recommended for patients with recurrent or refractory TTP, at a dose of 1.0 g/kg/d for 2 days or 0.4 g/kg/d for 5 days. If necessary, it can be given repeatedly, but the therapeutic effect may not be as good as plasma exchange.	Class II; 78.8%
AHA	In patients with AHA who do not respond to immunosuppressive regimens, the administration of IVIG 1.0 g/kg/d for 2 days or 0.4 g/kg/d for 5 days is recommended IVIG has poor efficacy in the AHA, and therefore its use for the purpose of blocking autoantibodies to FVIII is not recommended.	Class II;78.8%
AIHA	High-dose IVIG can be used for life-threatening hemolysis or hemolysis for which other treatments are ineffective, and it is recommended that high-dose corticosteroids be given in combination with IVIG 1.0 g/kg/d for 2 days or 0.4 g/kg/d for 5 days as salvage therapy only in cases of severe or rapid hemolysis.	Class I; 93.9%
HG	When complications related to primary immunodeficiency, such as infection, occur in HG patients, prophylactic treatment IVIG infusion, IVIG 0.4 g/kg/dose, is used, recommended every 3 weeks typically for the remainder of the patient’s life.	Class II; 75.8%
Application of IVIG in the treatment of hematological tumors		
Chimeric antigen receptor T cell immunotherapy	After CAR-T cell therapy, the number of B lymphocytes and immunoglobulins level should be checked regularly, and IVIG infused at least once a month, at a dose of 0.4 g/kg/dose, until the immunoglobulins and B cells returned to normal range or 6 months after CAR-T cell therapy.	Class I; 90.9%
	Patients with serum IgG < 4 g/L and severe or recurrent infections after CAR-T cell therapy should continue to receive IVIG infusion at least once a month at a dose of 0.4 g/kg/dose until the risk factors are eliminated.	Class I; 90.9%
	If the serum IgG level is 4–6 g/L and there is still severe or recurrent infections after treatment, IVIG infusion should be given at least once a month at a dose of 0.4 g/kg/dose.	
	If the serum IgG is greater than 6 g/L and there are recurrent infections, it is recommended to further evaluate the levels of other types of immunoglobulins (IgG, IgA, IgM and IgE) and the number of B cells as well as specific antibody titers.	
Chemotherapy for acute leukemia	Individualized assessment of the benefits and risks of IVIG infusion in patients with AL is recommended, and after weighing the risks and costs associated with treatment, at least one IVIG infusion per month is recommended at IVIG 0.4 g/kg/dose, with a target serum IgG of 4 to 6 g/L, and in the presence of breakthrough infections, a serum IgG > 6 to 8 g/L. Serum IgG levels, specific antibody titers and infection pattern are used to guide the IVIG treatment.	Class II; 75.8%
	For AL patients with ineffective repeated platelet transfusion and obvious adverse reactions to blood transfusion, IVIG before transfusion can be recommended, at a dose of 0.2–0.4 g/kg/dose.	Class II; 78.8%
Treatment of multiple myeloma	For MM patients with serum IgG ≤4 g/L and severe or recurrent infections, at least one IVIG infusion of 0.4 g/kg/dose per month is recommended, and for MM patients after CAR-T cell therapy, at least one IVIG infusion of 0.4 g/kg/dose per month is recommended until 1 year after the end of CAR-T therapy.	Class II; 81.8%
Treatment of non-Hodgkin lymphoma	NHL patients need regular infusion of IVIG to enhance immunity and prevent infection during treatment, especially for NHL patients with IgG ≤ 4 g/L or recurrent infection or severe infection. The dose of IVIG is 0.4 g/kg/dose every 3–4 weeks.	Class II; 75.8%
Application of hematopoietic stem cell transplantation		
Application in post-transplant CMV infection	It is recommended that IVIG can be used prophylactically in patients with high risk for CMV activation in allo-HSCT, with 1 to 2 weekly infusions of IVIG, 5 g to 10 g/dose, up to 100 days post-transplantation.	Class II; 84.8%
Application in GVHD after transplantation	IVIG 0.5 g/kg per week from pre-transplantation −7 days to post-transplantation day 90 and 0.5 g/kg per month from post-transplantation day 90 to 1 year post-transplantation is recommended for those at high risk for GVHD when mismatched with allo-HSCT.	Class II; 84.8%
Application in post-transplant bacterial infections	IVIG 0.5 g/kg per week is recommended for patients with serum IgG <4 g/L from 7 days pretransplant to 90 days posttransplant, and 0.5 g/kg per month from 90 days posttransplant to 1 year posttransplant, so as to prevent serious bacterial infection. Serum IgG concentration should be monitored every 2 weeks, and individualized treatment should be carried out according to serum IgG level and infection.	Class II; 75.8%
